# Supplementary material for: Architecture and autoinhibitory mechanism of the plasma membrane Na+/H+ antiporter SOS1 in Arabidopsis
Source: Nat Commun. 2023 Jul 26;14:4487. doi: 10.1038/s41467-023-40215-y (PMC10372031; doi:10.1038/s41467-023-40215-y)
Supplement: Supplementary file 1 — Supplementary information [file 41467_2023_40215_MOESM1_ESM.pdf]

Supplementary Information for

Architecture and autoinhibitory mechanism of the plasma membrane Na^+/H^+ antiporter SOS1 in *Arabidopsis*

Yuhang Wang[1,2,3], Chengcai Pan[4], Qihao Chen[1,2,3], Qing Xie[4], Yiwei Gao[1,2,3], Lingli He[1,2,3], Yue Li[1,2,3], Yanli Dong[1,2,3], Xingyu Jiang[4]*, Yan Zhao[1,2,3]*

[1]National Laboratory of Biomacromolecules, CAS Center for Excellence in Biomacromolecules, Institute of Biophysics, Chinese Academy of Sciences, Beijing 100101, China

[2]State Key Laboratory of Brain and Cognitive Science, Institute of Biophysics, Chinese Academy of Sciences, 15 Datun Road, Beijing, 100101, China

[3]College of Life Sciences, University of Chinese Academy of Sciences, Beijing 100049, China

[4]National Center for Technology Innovation of Saline-Alkali tolerant Rice/College of Coastal Agricultural Sciences, Guangdong Ocean University, Zhanjiang 524088, China

Correspondence emails: jiangxingyuhu@163.com (X.J.) and zhaoy@ibp.ac.cn (Y.Z.)

This PDF file includes Supplementary Figure. 1–15 and Supplementary Table 1–3.

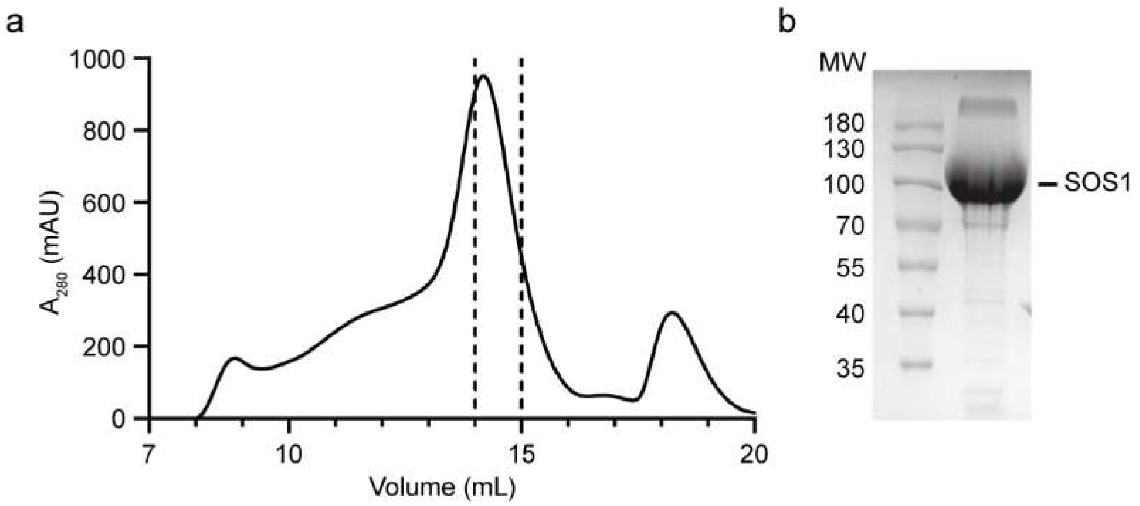

Supplementary Figure 1. Purification of SOS1. **a**, Size-exclusion chromatogram of SOS1. The peak fraction (marked within black dashed lines) was collected and concentrated for cryo-EM analysis. **b,** SDS-PAGE followed by Coomassie blue staining of the purified SOS1 samples. MW, molecular weight marker (kDa). The experiments were repeated independently for more than three times with identical results. Source data are provided as a Source Data file.

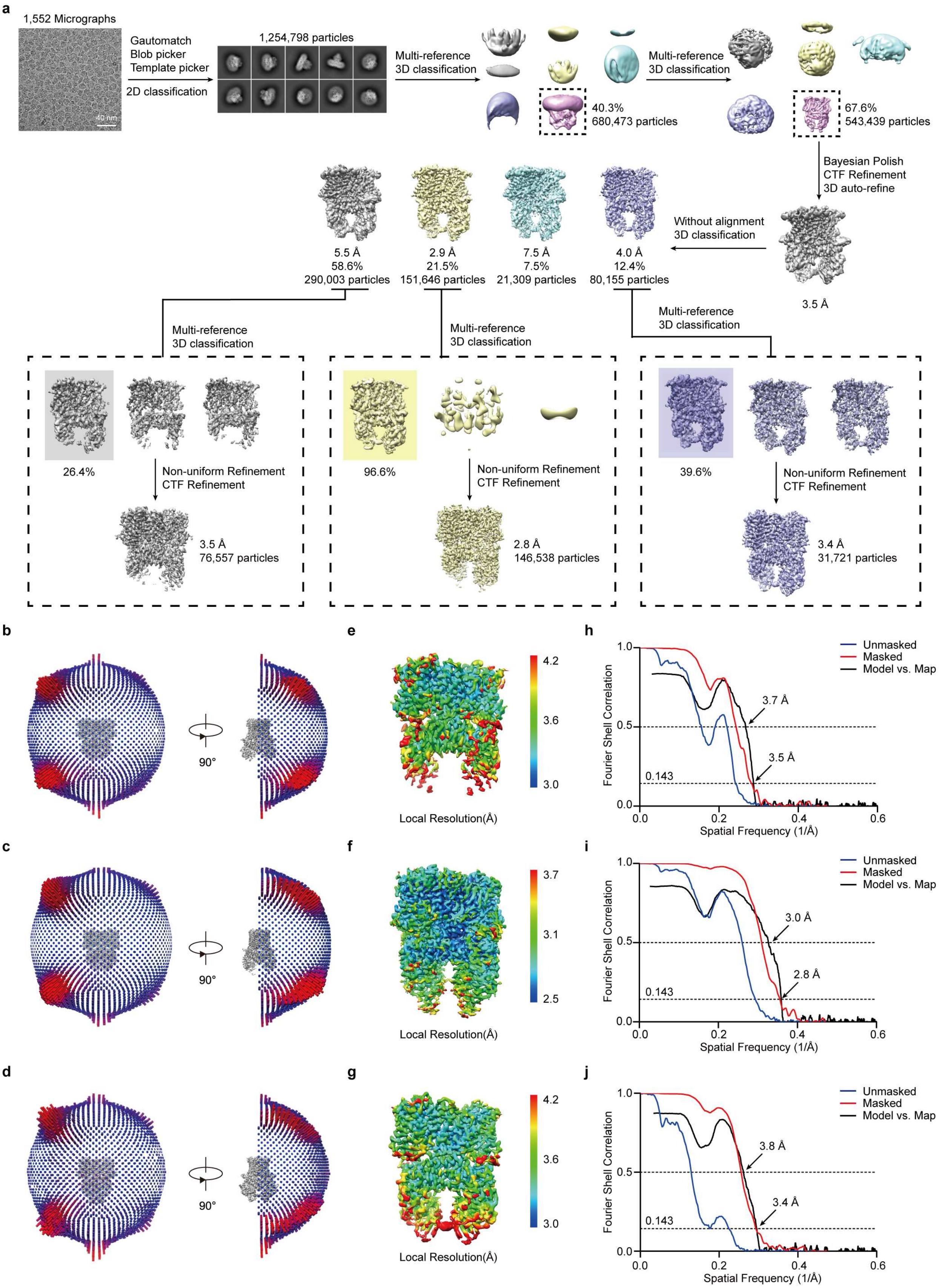

Supplementary Figure 2. Single-particle cryo-EM analysis of SOS1. **a,** Flow chart of the process of structure determination. A total of 1,552 movie stacks were collected (Bar = 40

nm). A representative motion-corrected micrograph of this dataset is shown here. Particles were picked using Gautomatch, Blob picker and template picker, and were then subjected to several rounds of 3D classification, *Bayesian* Polish, CTF refinement and 3D-auto refinement. The final map was reported at 3.5 Å, 2.8 Å and 3.4 Å according to the GSFSC criterion. **b-d,** The angular distribution of the particles that contributes to the final reconstitution. The length of each spike indicates the number of particles in the designated orientation. **e-g,** Local resolution of the cryo-EM density map. Variations in local resolution are color-coded from blue to red as indicated. **h-j,** The half-map (red) and model-map (black) *Fourier* shell correlation (FSC) curves of SOS1.

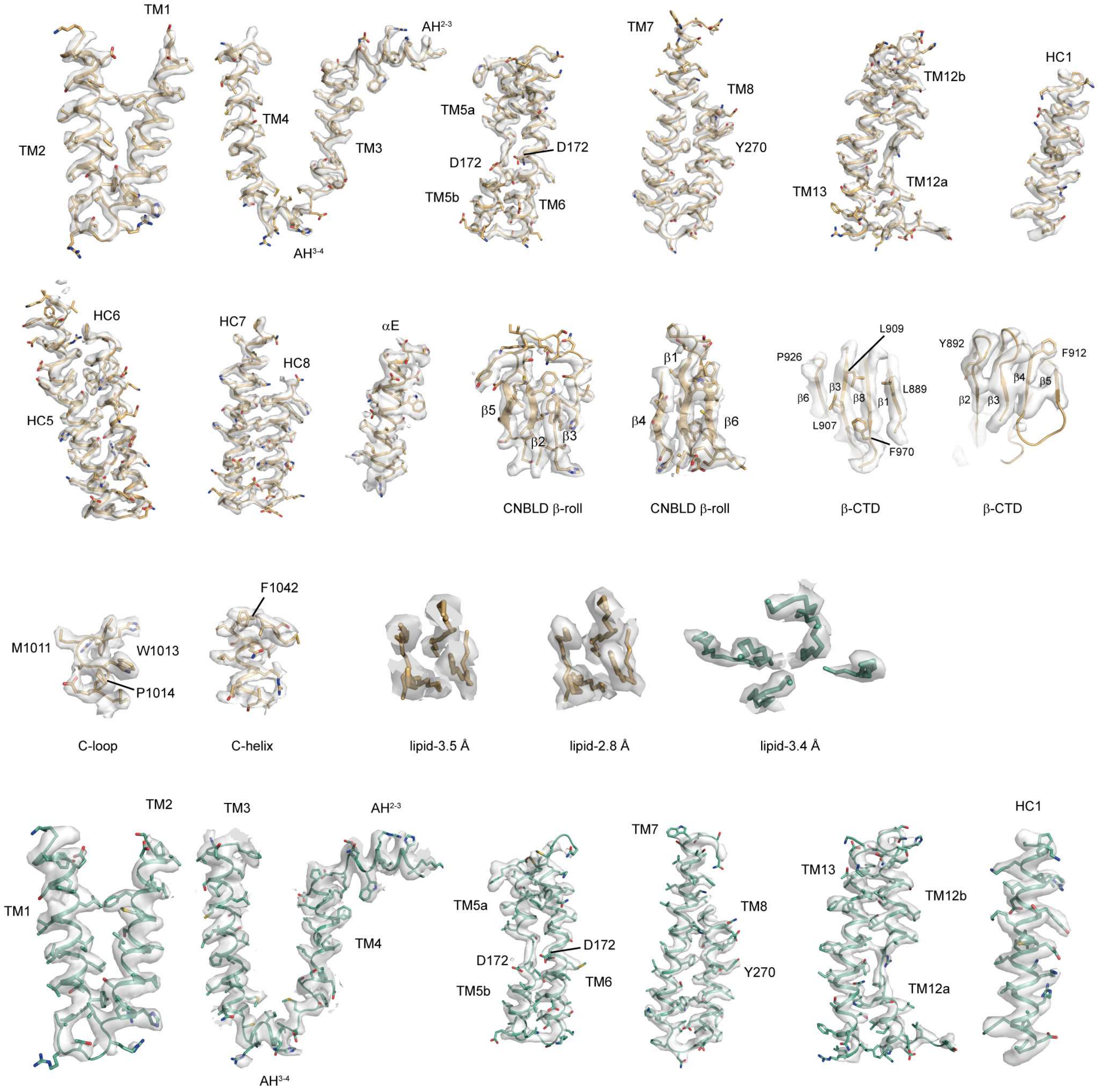

Supplementary Figure 3. Representative local cryo-EM densities for the SOS1 (yellow) and SOS1^{expand} (green) and lipid molecules.

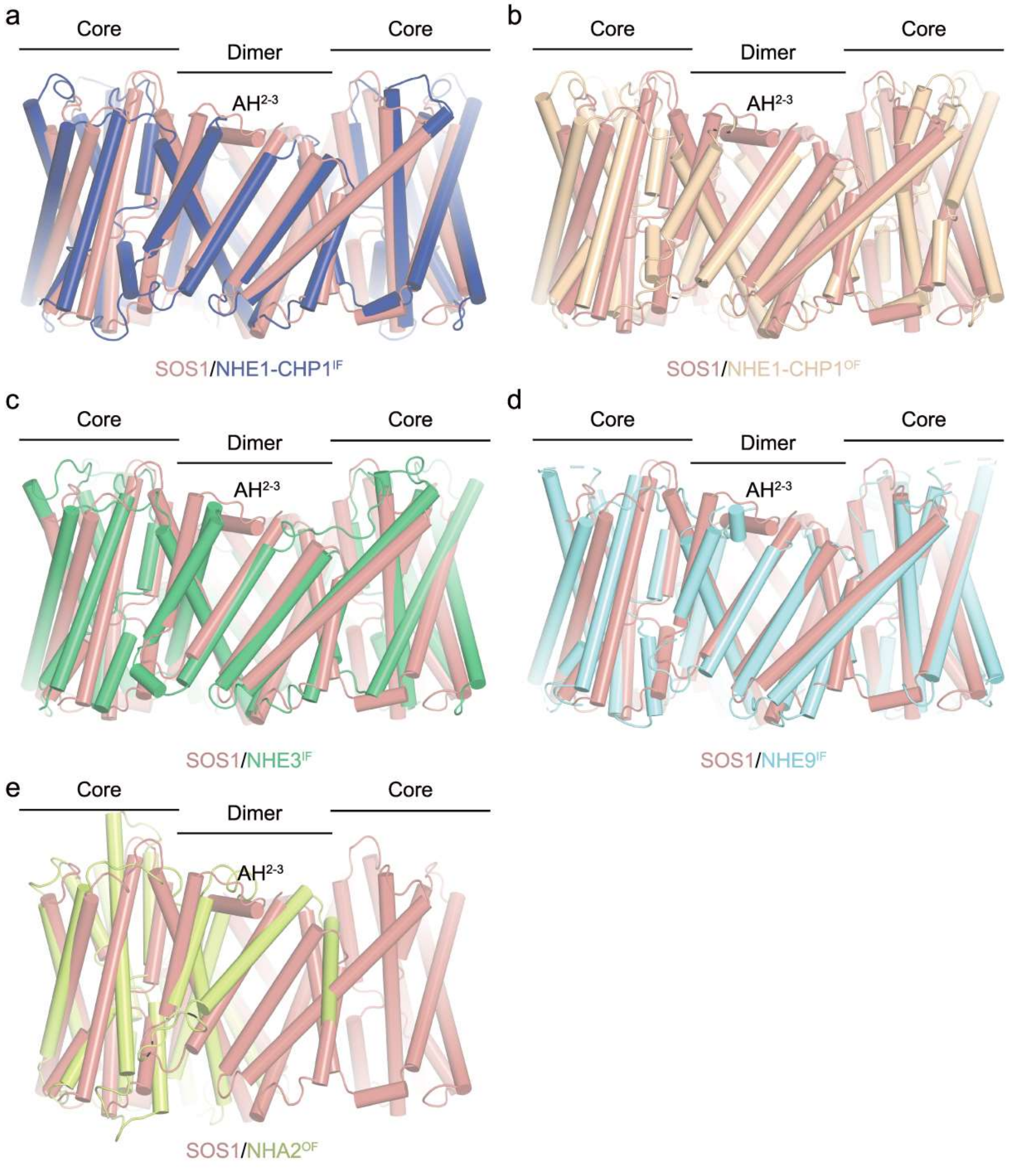

Supplementary Figure 4. Structural comparison of the transmembrane regions of SOS1 (residue 32-450) with inward-facing human NHE1-CHP1^{IF} (PDB ID: 7DSV, residue 98-506) **(a)**, outward-facing human NHE1-CHP1^{OF} (PDB ID: 7DSX, residue 98-506) **(b)**, inward-facing human NHE3^{IF} (PDB ID: 7X2U, residue 40-465) **(c)**, inward-facing horse NHE9 (PDB ID: 6Z3Y, residue 23-489) **(d)** and outward-facing bison NHA2 (PDB ID: 7P1I, residue 79-517) **(e),** using entire TMD as the reference. The helices are displayed as cylinders. The RMSD

values are 2.4 Å, 3.2 Å, 2.7 Å, 3.0 Å and 3.3 Å, respectively. The structural comparison with NHA2 (14TMs) is present in monomeric form for the existence of the extra N-terminal helix.

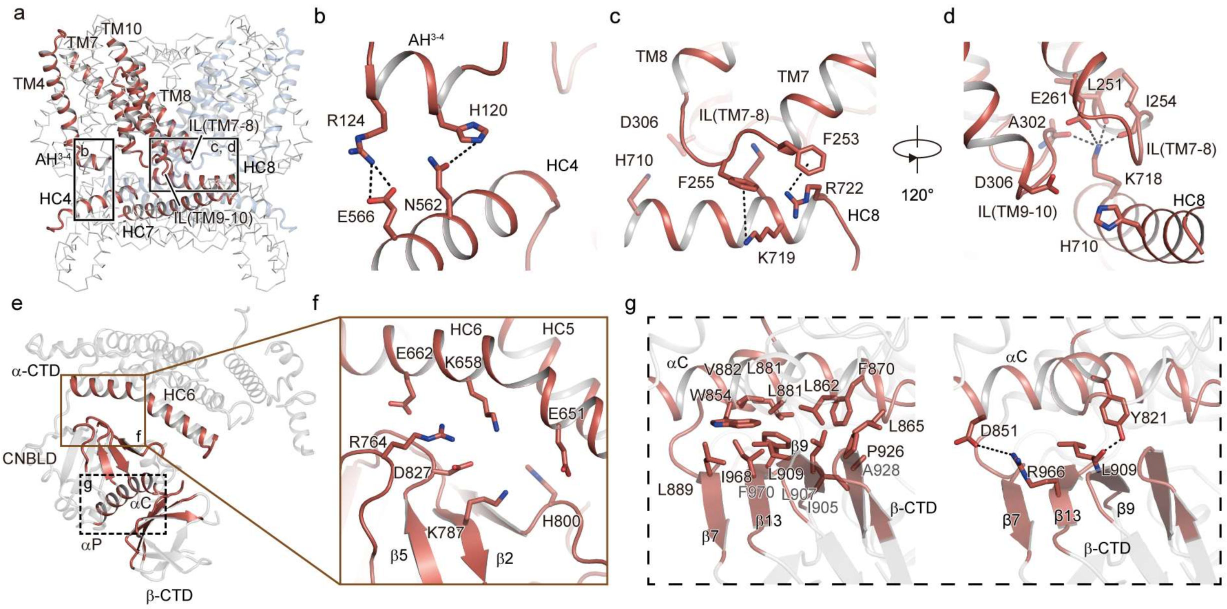

Supplementary Figure 5. Interdomain interactions within a protomer. **a–d,** Overall (**a**) and zoomed-in (**b**–**d**) views of the interaction interfaces between TMD and α-CTD. **a,** The secondary elements involved in domain interactions are shown as colored cartoon and labeled. **b,** Polar interactions of H120–N562 and R124–E566 between AH$^{3-4}$ and HC4. **c,** Cation-π interactions between cationic residues (K719, R722) on HC8 and aromatic residues (F253, F255) on IL(TM7-8). **d,** The sidechain of K718 on HC8 is inserted into the space between IL(TM7-8) and IL(TM9-10), and the residues which are connected to K718 by hydrogen bonds are labeled. Panel (**c**) and (**d**) differ by 120°. **e,** Overall view of interaction interfaces among the α-CTD, CNBLD and β-CTD. **f,** Extensive electrostatic interactions between α-CTD and CNBLD. Charged residues involved in the electrostatic interactions are shown. **g,** Hydrophobic interactions (left) and polar interactions (right) at the surface of CNBLD and β-CTD. Sidechains of residues involved in the interactions are displayed as sticks. Dashed lines represent salt bridges and hydrogen bonds.

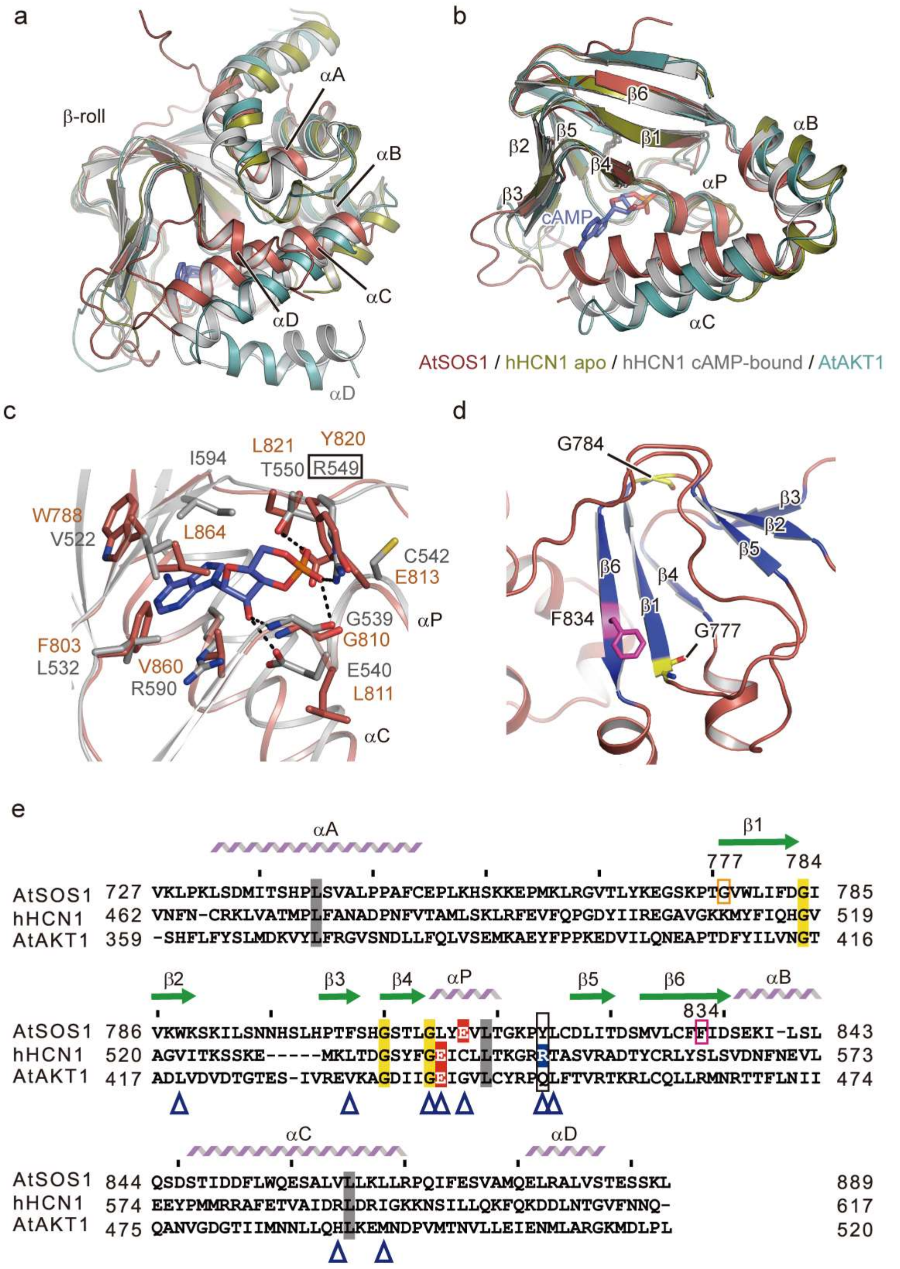

Supplementary Figure 6. The CNBLD of SOS1. **a,** Superposition of CNBLD from SOS1 (orange) (residues 758-866) with CNBD from human HCN1 in the ligand-free state (deepolive, PDB ID: 5U6O) (residues 491-586), human HCN1 in the cAMP-bound state (gray, PDB ID: 5U6P) (residues 491-594) and *Arabidopsis thaliana* AKT1 in the ligand-free state (teal, PDB ID: 7WSW) (residues 388-497) based on Cα atoms of the β-roll. **b,** Superposition of the cAMP-binding region. cAMP molecule in the HCN1 structure was shown in sticks. c, Structural comparison of the cAMP-binding region. Residues directly interacting with cAMP in the CNBD of HCN1 channel and the corresponding residues in the CNBLD of SOS1 are

shown in sticks. The dashed lines show the salt-bridge and polar interactions between phosphoribose of cAMP and residues on the αP and loop between αP and β5. **d,** β-roll of the CNBLD. Residues G777 and G784 are shown in sticks and highlighted in yellow. F834, which is form “mortise-tenon” joint with G777 is highlighted in pink. **e,** Sequence alignment of the CNBLD region. Secondary structures of SOS1 are indicated above the sequence. Positively and negatively charged residues are highlighted in red and blue, respectively. The conserved glycine residues are highlighted in yellow. The glycine-aromatic residue pairs in SOS1 are marked with boxes. Residues which directly interact with the cAMP displayed in (**b**) are marked with blue triangles.

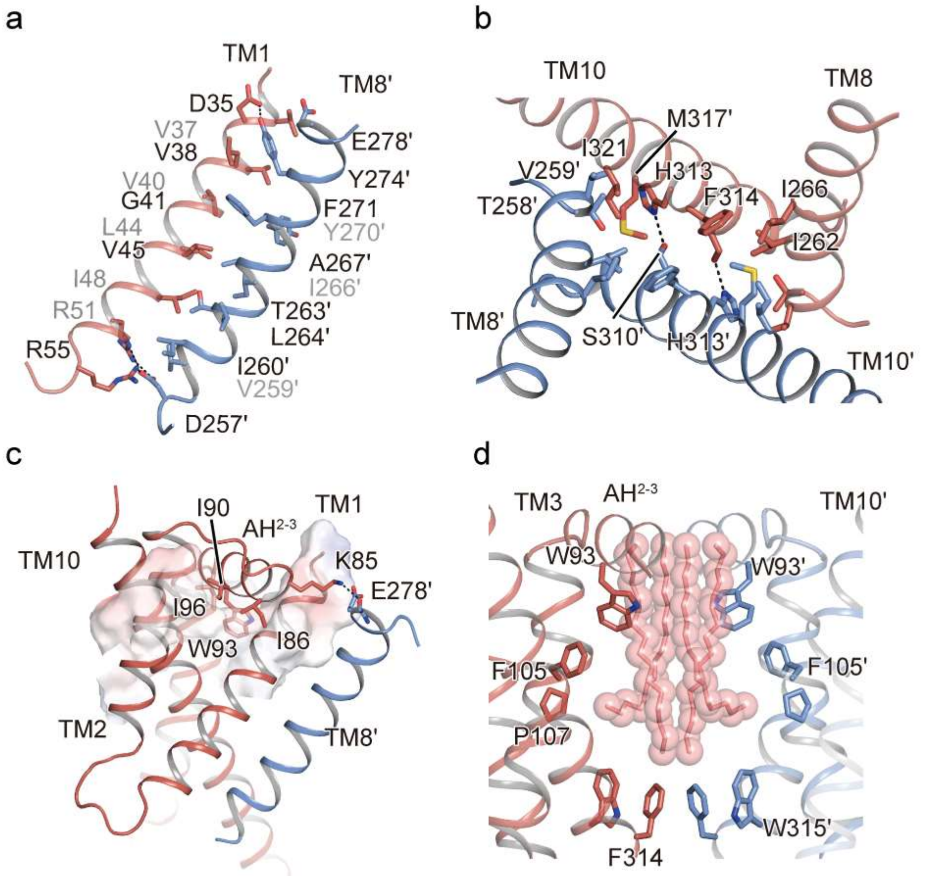

Supplementary Figure 7. TMD dimerization interfaces of SOS1. **a,** Hydrophobic interactions between TM1 from one protomer and TM8′ from another protomer. Sidechains of residues in dimer contact are showed in sticks. **b,** TM10 and TM8 helices from two protomers form a 2-fold symmetrical hydrophobic cluster. Hydrophobic residues are shown as sticks. **c,** Polar interaction between AH$^{2-3}$ and TM8'. The interactions are indicated by black dotted lines. Residues (I86, I90, W93 and I96) on AH$^{2-3}$ involved in hydrophobic interactions with TM1 and TM10 are shown as sticks. **d,** Hydrophobic cavity between the two dimerization domains. Aromatic amino acid residues are shown as sticks. Helices are shown as cartoon. Putative lipids (salmon) within the cavity are shown as sticks overlaid with transparent red spheres.

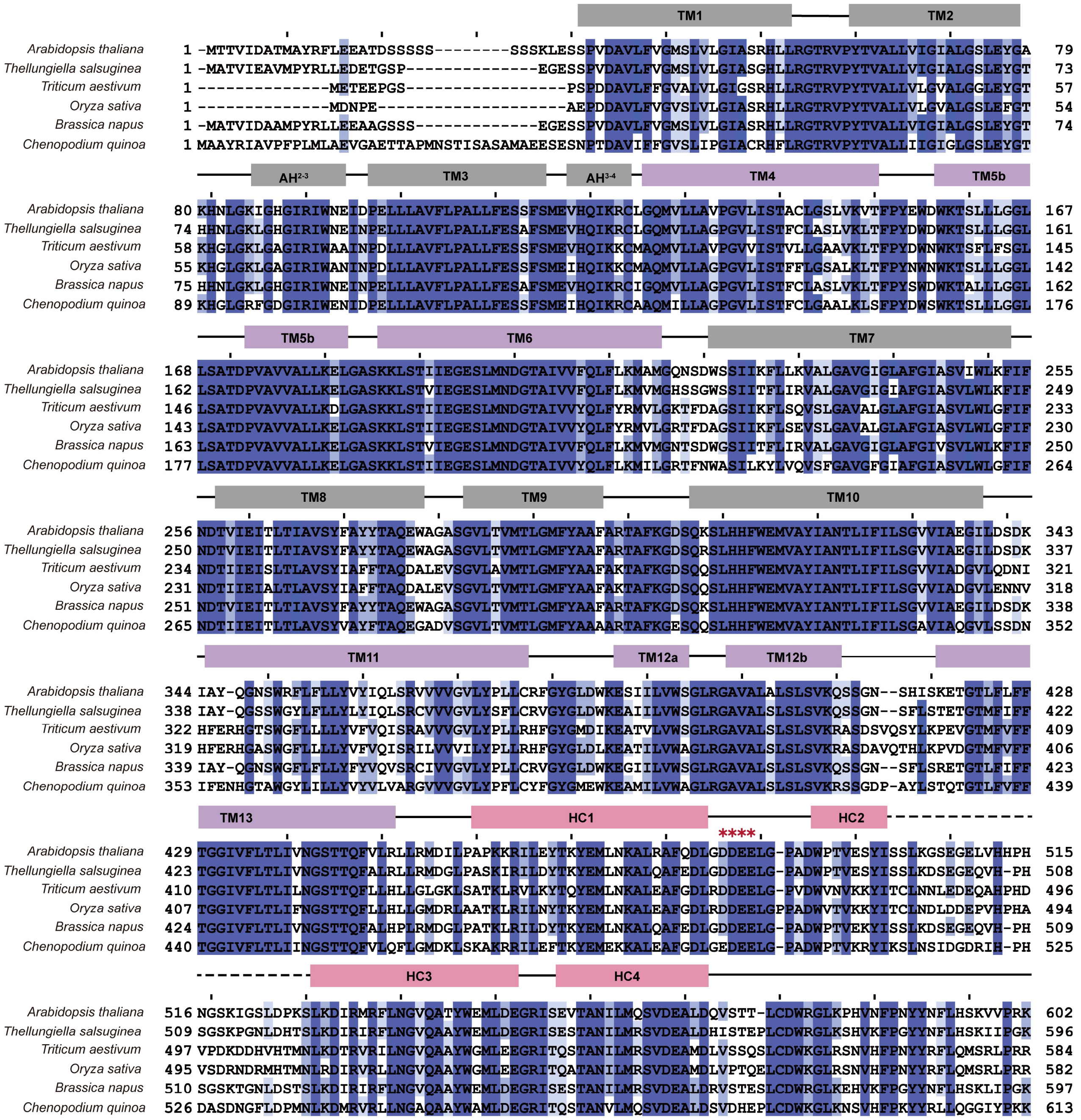

TM1
TM2
Arabidopsis thaliana 1 -MTTVIDATMAYRFLEEATDSSSSS--------SSSKLESSPVDAVLFVGMSLVLGIASRHLLRGTRVPYTVALLVIGIALGSLEYGA 79
Thellungiella salsuginea 1 -MATVIEAVMPYRLLEDETGSP--------------EGESSPVDAVLFVGMSLVLGIASGHLLRGTRVPYTVALLVIGIALGSLEYGT 73
Triticum aestivum 1 --------------METEEPGS-----------------PSPDDAVLFFGVALVLGIGSRHLLRGTRVPYTVALLVLGVALGGLEYGT 57
Oryza sativa 1 --------------MDNPE--------------------AEPDDAVLFVGVSLVLGIASRHLLRGTRVPYTVALLVLGVALGSLEFGT 54
Brassica napus 1 -MATVIDAAMPYRLLEEAAGSSS-------------EGESSPVDAVLFVGMSLVLGIASRHLLRGTRVPYTVALLVIGIALGSLEYGT 74
Chenopodium quinoa 1 MAAYRIAVPFPLMLAEVGAETTAPMNSTISASAMAEESESNPTDAVIFFGVSLIPGIACRHFLRGTRVPYTVALLIIGIGLGSLEYGT
AH2-3
TM3
AH3-4
TM4
TM5b
Arabidopsis thaliana 80 KHNLGKIGHGIRIWNEIDPELLLAVFLPALLFESSFSMEVHQIKRCLGQMVLLAVPGVLISTACLGSLVKVTFPYEWDWKTSLLLGGL 167
Thellungiella salsuginea 74 HHNLGKLGHGIRIWNEINPELLLAVFLPALLFESAFSMEVHQIKRCLGQMVLLAGPGVLISTFCLASLVKLTFPYDWDWKTSLLLGGL 161
Triticum aestivum 58 KHGLGKLGAGIRIWAAINPDLLLAVFLPALLFESSFSMEVHQIKKCMAQMVLLAVPGVVISTVLLGAAVKLTFPYDWNWKTSFLFSGL 145
Oryza sativa 55 KHGLGKLGAGIRIWANINPDLLLAVFLPALLFESSFSMEIHQIKKCMAQMVLLAGPGVLISTFFLGSALKLTFPYNWNWKTSLLLGGL 142
Brassica napus 75 HHNLGKLGHGIRIWNEINPELLLAVFLPALLFESAFSMEVHQIKRCIGQMVLLAGPGVLISTFCLASLVKLTFPYSWDWKTALLLGGL 162
Chenopodium quinoa 89 KHGLGRFGDGIRIWENIDPELLLAVFLPALLFESSFSMEIHQIKRCAAQMILLAGPGVLISTFCLGAALKLSFPYDWSWKTSLLLGGL 176
TM5b
TM6
TM7
Arabidopsis thaliana 168 LSATDPVAVVALLKELGASKKLSTIIEGESLMNDGTAIVVFQLFLKMAMGQNSDWSSIIKFLLKVALGAVGIGLAFGIASVIWLKFIF 255
Thellungiella salsuginea 162 LSATDPVAVVALLKELGASKKLSTVIEGESLMNDGTAIVVFQLFLKMVMGHSSGWSSIITFLIRVALGAVGIGIAFGIASVLWLKFIF 249
Triticum aestivum 146 LSATDPVAVVALLKDLGASKKLSTIIEGESLMNDGTAIVVYQLFYRMVLGKTFDAGSIIKFLSQVSLGAVALGLAFGIASVLWLGFIF 233
Oryza sativa 143 LSATDPVAVVALLKELGASKKLSTIIEGESLMNDGTAIVVYQLFYRMVLGRTFDAGSIIKFLSEVSLGAVALGLAFGIASVLWLGFIF 230
Brassica napus 163 LSATDPVAVVALLKELGASKKLSTVIEGESLMNDGTAIVVFQLFLKMVMGNTSDWGSIITFLIRVALGAVGIGLAFGIVSVLWLKFIF 250
Chenopodium quinoa 177 LSATDPVAVVALLKELGASKKLSTIIEGESLMNDGTAIVVYQLFLKMILGRTFNWASILKYLVQVSFGAVGFGIAFGIASVLWLGFIF 264
TM8
TM9
TM10
Arabidopsis thaliana 256 NDTVIEITLTIAVSYFAYYTAQEWAGASGVLTVMTLGMFYAAFARTAFKGDSQKSLHHFWEMVAYIANTLIFILSGVVIAEGILDSDK 343
Thellungiella salsuginea 250 NDTVIEITLTIAVSYFAYYTAQEWAGASGVLTVMTLGMFYAAFARTAFKGDSQRSLHHFWEMVAYIANTLIFILSGVVIAEGILDSDK 337
Triticum aestivum 234 NDTIIEISLTLAVSYIAFFTAQDALEVSGVLAVMTLGMFYAAFAKTAFKGDSQQSLHHFWEMVAYIANTLIFILSGVVIADGVLQDNI 321
Oryza sativa 231 NDTIIEIALTLAVSYIAFFTAQDALEVSGVLTVMTLGMFYAAFAKTAFKGDSQQSLHHFWEMVAYIANTLIFILSGVVIADGVLENNV 318
Brassica napus 251 NDTVIEITLTIAVSYFAYYTAQEWAGASGVLTVMTLGMFYAAFARTAFKGDSQKSLHHFWEMVAYIANTLIFILSGVVIAEGILDSDK 338
Chenopodium quinoa 265 NDTIIEITLTLAVSYVAYFTAQEGADVSGVLTVMTLGMFYAAAARTAFKGESQQSLHHFWEMVAYIANTLIFILSGAVIAQGVLSSDN 352
TM11
TM12a
TM12b
Arabidopsis thaliana 344 IAY-QGNSWRFLFLLYVYIQLSRVVVVGVLYPLLCRFGYGLDWKESIILVWSGLRGAVALALSLSVKQSSGN--SHISKETGTLFLFF 428
Thellungiella salsuginea 338 IAY-QGSSWGYLFLLYLYIQLSRCVVVGVLYSFLCRVGYGLDWKEAIILVWSGLRGAVALSLSLSVKQSSGN--SFLSTETGTMFIFF 422
Triticum aestivum 322 HFERHGTSWGFLLLLYVFVQISRAVVVGVLYPLLRHFGYGMDIKEATVLVWSGLRGAVALSLSLSVKRASDSVQSYLKPEVGTMFVFF 409
Oryza sativa 319 HFERHGASWGFLLLLYVFVQISRILVVVILYPLLRHFGYGLDLKEATILVWAGLRGAVALSLSLSVKRASDAVQTHLKPVDGTMFVFF 406
Brassica napus 339 IAY-QGNSWGFLFLLYFYVQVSRCIVVGVLYPLLCRVGYGLDWKEGIILVWSGLRGAVALSLSLSVKQSSGN--SFLSRETGTLFIFF 423
Chenopodium quinoa 353 IFENHGTAWGYLILLYVYVLVARGVVVGVLYPFLCYFGYGMEWKEAMILVWAGLRGAVALSLSLSVKRSSGDP-AYLSTQTGTLFVFF 439
TM13
HC1
HC2
****
Arabidopsis thaliana 429 TGGIVFLTLIVNGSTTQFVLRLLRMDILPAPKKRILEYTKYEMLNKALRAFQDLGDDEELG-PADWPTVESYISSLKGSEGELVHHPH 515
Thellungiella salsuginea 423 TGGIVFLTLIVNGSTTQFALRLLRMDGLPASKIRILDYTKYEMLNKALQAFEDLGDDEELG-PADWPTVESYISSLKDSEGEQVH-PH 508
Triticum aestivum 410 TGGIVFLTLILNGSTTQFLLHLLGLGKLSATKLRVLKYTQYEMLNKALEAFGDLRDDEELG-PVDWVNVKKYITCLNNLEDEQAHPHD 496
Oryza sativa 407 TGGIVFLTLIFNGSTTQFLLHLLGMDRLAATKLRILNYTKYEMLNKALEAFGDLRDDEELGPPADWVTVKKYITCLNDLDDEPVHPHA 494
Brassica napus 424 TGGIVFLTLIVNGSTTQFALHPLRMDGLPATKLRILDYTKYEMLNKALQAFEDLGDDEELG-PADWPTVEKYISSLKDSEGEQVH-PH 509
Chenopodium quinoa 440 TGGIVFLTLIINGSTTQFVLQFLGMDKLSKAKRRILEFTKYEMEKKALEAFGDLGEDEELG-PADWPTVKRYIKSLNSIDGDRIH-PH 525
HC3
HC4
Arabidopsis thaliana 516 NGSKIGSLDPKSLKDIRMRFLNGVQATYWEMLDEGRISEVTANILMQSVDEALDQVSTT-LCDWRGLKPHVNFPNYYNFLHSKVVPRK 602
Thellungiella salsuginea 509 SGSKPGNLDHTSLKDIRIRFLNGVQAAYWEMLDEGRISESTANILMRSVDEALDHISTEPLCDWRGLKSHVKFPGYYNFLHSKIIPGK 596
Triticum aestivum 497 VPDKDDHVHTMNLKDTRVRILNGVQAAYWGMLEEGRITQSTANILMRSVDEAMDLVSSQSLCDWKGLRSNVHFPNYYRFLQMSRLPRR 584
Oryza sativa 495 VSDRNDRMHTMNLRDIRVRLLNGVQAAYWGMLEEGRITQATANILMRSVDEAMDLVPTQELCDWKGLRSNVHFPNYYRFLQMSRLPRR 582
Brassica napus 510 SGSKTGNLDSTSLKDIRIRFLNGVQAAYWEMLDEGRISESTANILMRSVDEALDRVSTESLCDWRGLKEHVKFPGYYNFLHSKLIPGK 597
Chenopodium quinoa 526 DASDNGFLDPMNLKDMRVRLLNGAQAAYWAMLDEGRITQSTANVLMQSVDEALDSVDHEPLCDWKGLKNSVHFPKYYRLLQGGIYPKK 613

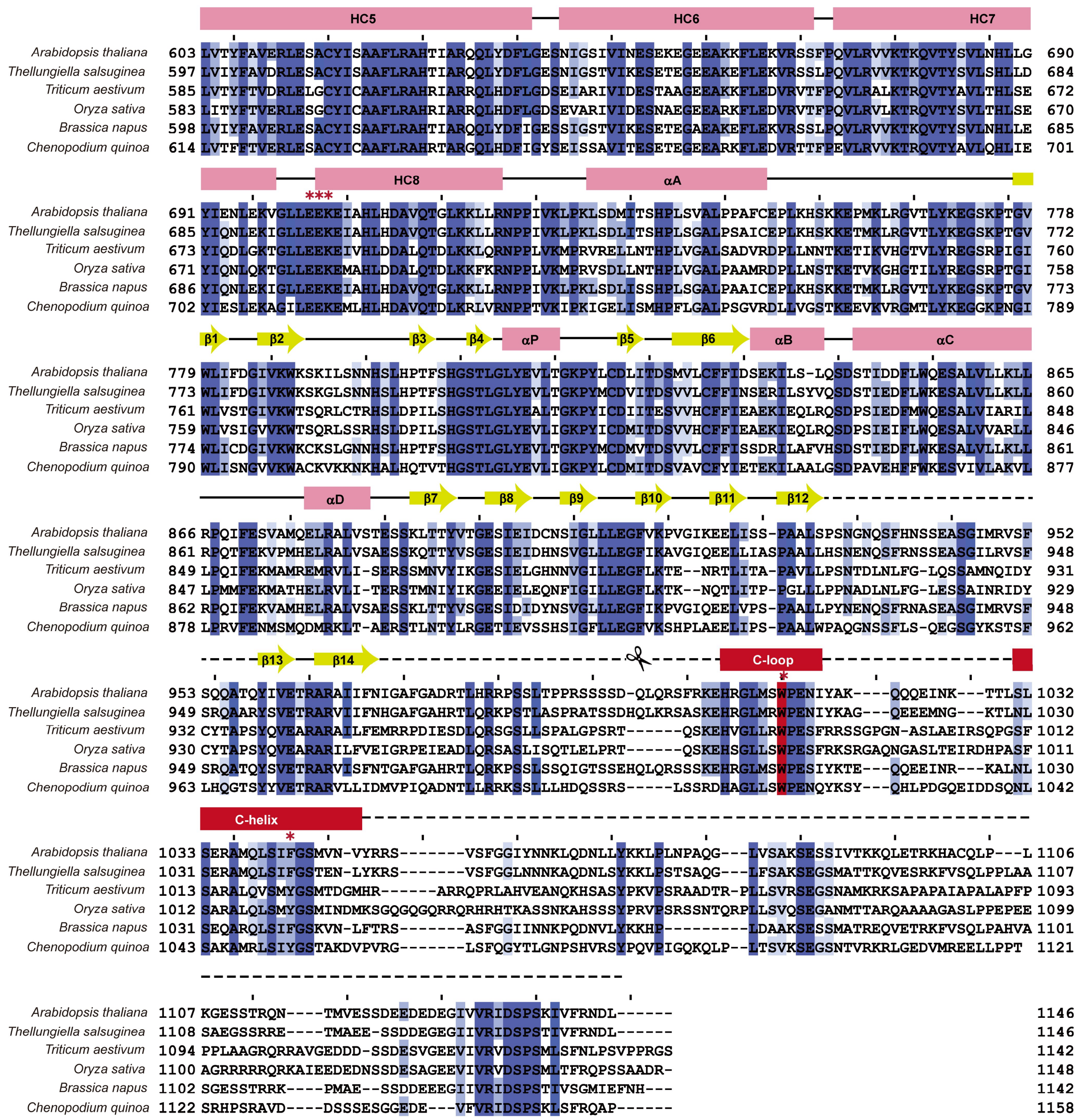

Supplementary Figure 8. Sequence alignment of SOS1 proteins. Secondary structural elements of *Arabidopsis thaliana* are marked above the sequence alignment. Unmodeled segments are represented as dashed lines. Helices of the dimerization domain, core domain, and cytoplasmic region are colored in gray, violet, and pink, respectively. The C-loop and C-helix are colored in red. Sheets are colored in yellow. Mutation sites are marked with red

asterisks. Conservative residues among these proteins are highlighted in blue with gradients representing conservative degree. The truncated site for SOS1$^{\Delta998}$ is marked with a scissor.

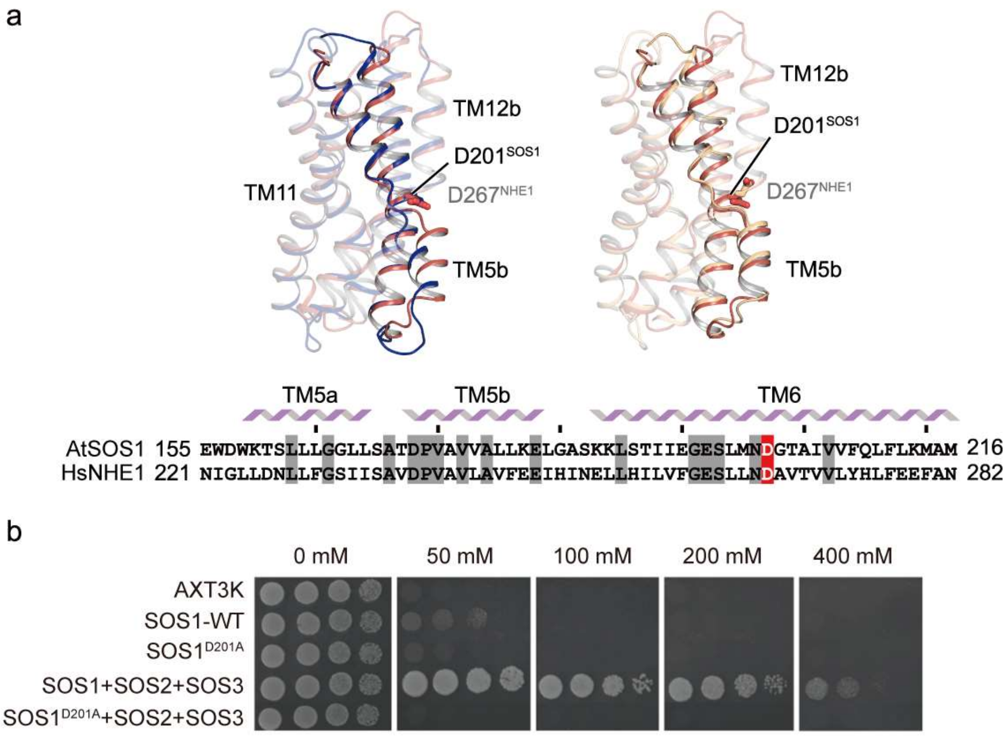

Supplementary Figure 9. Structure comparison between the TMD of SOS1 and NHE1-CHP1. **a,** The superimposed core domains of occluded SOS1 and inward-facing NHE1-CHP1^{IF} (blue), outward-facing NHE1-CHP1^{OF} (yellow), shown in cartoon. TM5 and TM6 were highlighted and the sequence alignment was shown below. Conserved residues are shown in gray and the aspartic acid on TM6 is highlighted in red. Secondary structures are indicated above the sequence. **b,** SOS1^{D201A} mutant was transformed in strain AXT3K, with and without the coexpression of p414-SOS2-SOS3, and compared with wide-type SOS1 in AP medium with the indicated concentrations of NaCl.

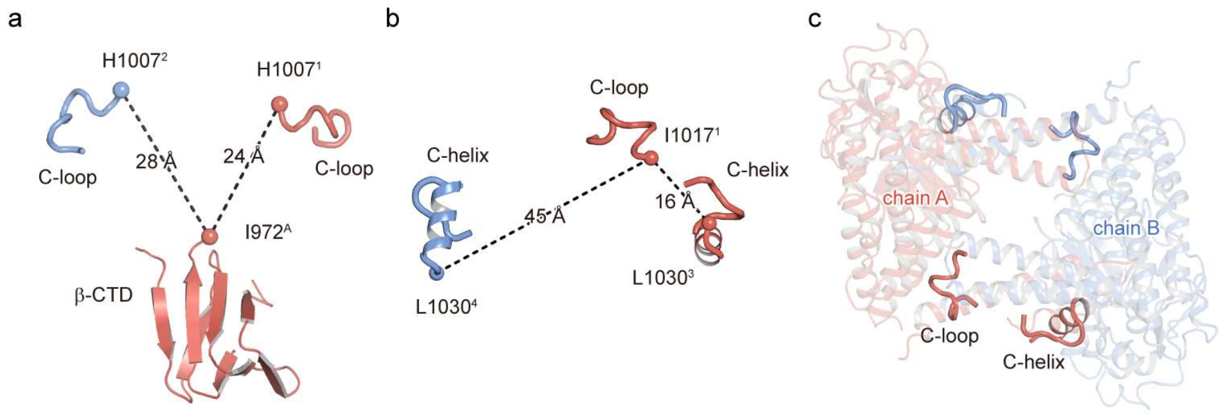

Supplementary Figure 10. C-loop and C-helix are swapped between the two monomers of SOS1. **a,** The distances between I972^{A} (the last residue of β-CTD) to H1007^{1} (the first residue of C-loop) or H1007^{2} are about 24 Å and 28 Å, respectively. **b,** The distances between I1017^{1} (the last residue of C-loop) and L1030^{3} (the first residue of C-helix) and between I1017^{1} and L1030^{4} are 16 Å and 45 Å, respectively. **c,** The swapped C-loop and C-helix that viewed from the extracellular side.

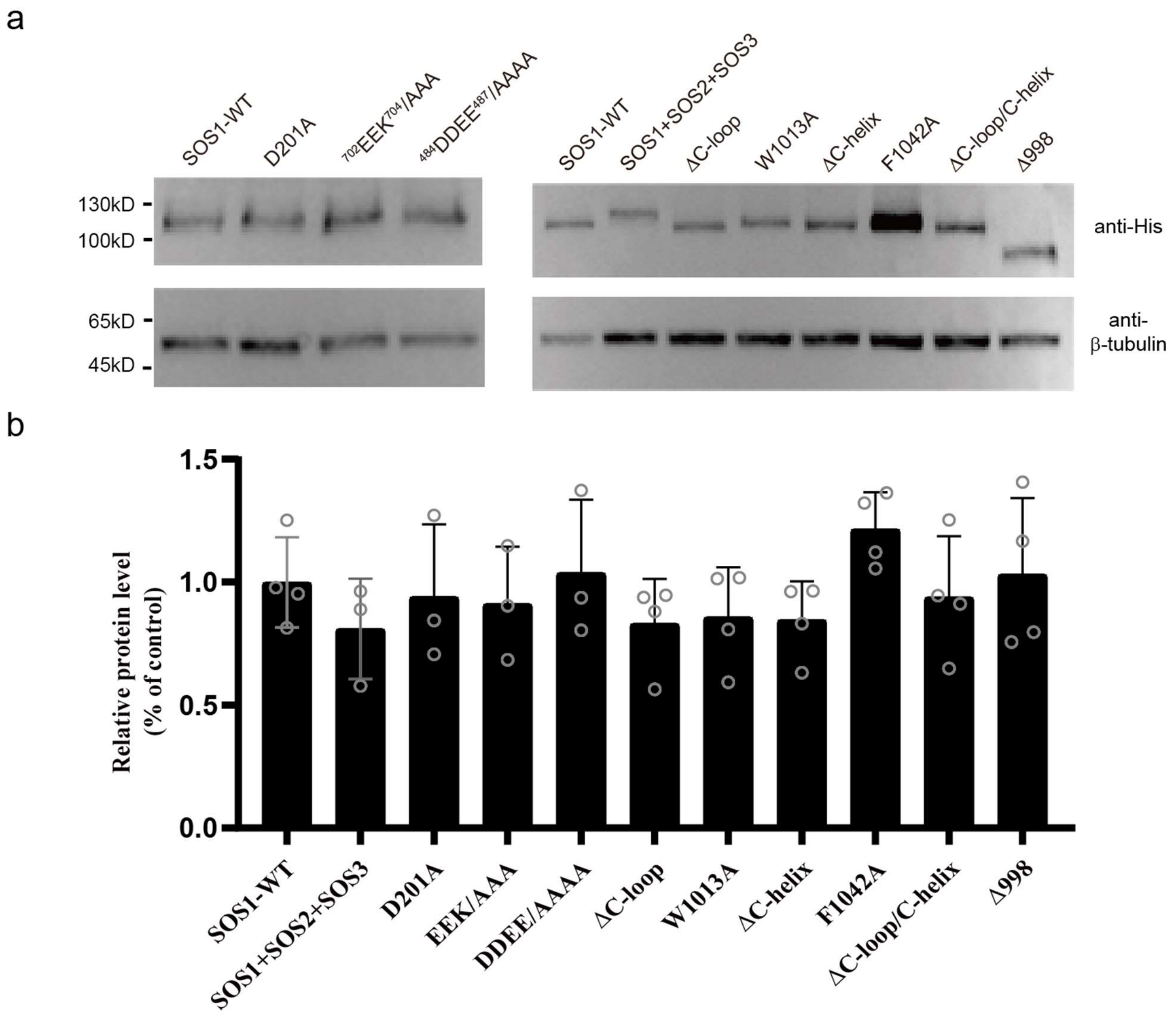

Supplementary Figure 11. Protein expression analysis SOS1 and derived mutants in yeasts. Western blot (**a**) and quantification (**b**) of wild-type and mutated SOS1 in transgenic yeast against the C-terminal His tag. Antibody against β-tubulin was used for the loading control. The levels of mutants were normalized to β-tubulin and presented as the relative expression levels to the wildtype SOS1. Data are obtained from densitometric scans by ImageJ and presented as means ± SEM of three independent experiments (SOS1+SOS2+SOS3, D201A, EEK/AAA and DDEE/AAAA) or four independent experiments (SOS1-WT, ΔC-loop, W1013A, ΔC-helix, F1042A, ΔC-loop/C-helix and Δ998). Each symbol represents a single data value. Source data are provided as a Source Data file.

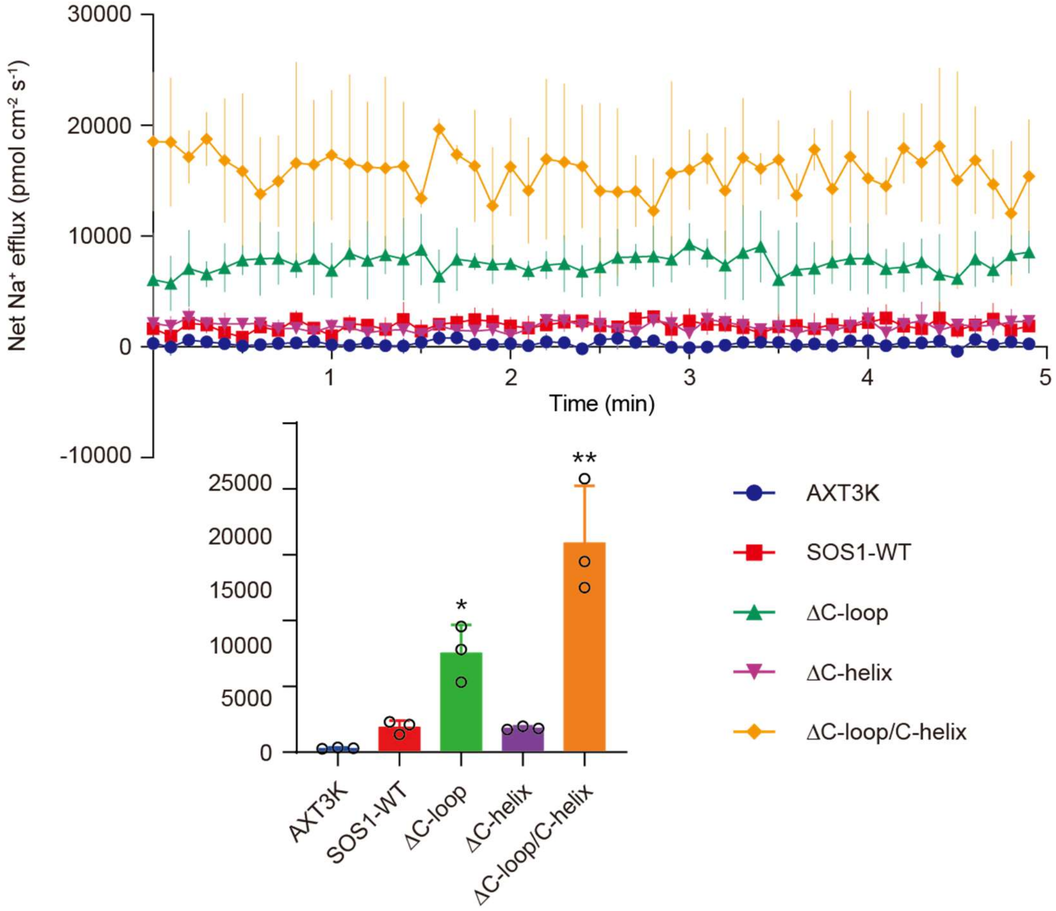

Supplementary Figure 12. Net Na^+ effluxes in yeast strains measured by NMT assay. Yeast strains expressing empty pYPGE15 vector, SOS1-WT, $SOS1^{\Delta C\text{-loop}}$, $SOS1^{\Delta C\text{-helix}}$, and $SOS1^{\Delta C\text{-loop/C-helix}}$ were subjected to ion fluxes measurements using NMT assay for 5 min. Each point represents the mean of three individual samples and the bars denote the standard error of the mean. The column chart shows the mean efflux rates of Na^+ within the measuring period. Each symbol represents a single measurement. Asterisks indicate significant differences compared with SOS1-WT. Statistical significance was determined by two-side and unpaired t-test, without making any adjustments for multiple comparisons (*$p < 0.05$; **$p < 0.01$). *P* value, SOS1-WT vs. ΔC-loop, 0.0109; SOS1-WT vs. ΔC-loop/C-helix, 0.0050. Source data are provided as a Source Data file.

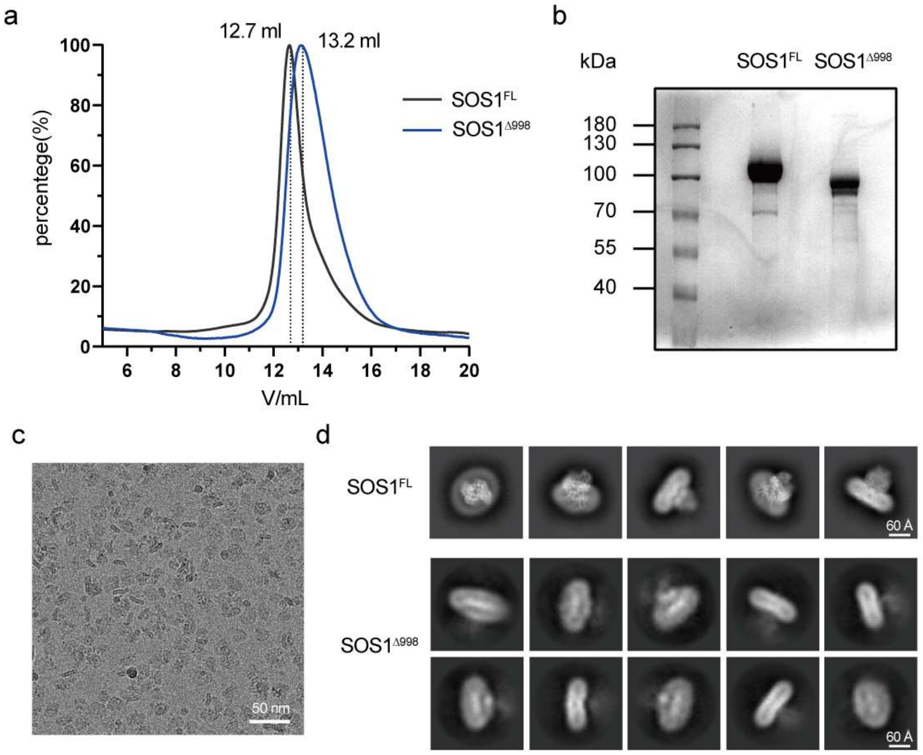

Supplementary Figure 13. Purification and cryo-EM analysis of samples for structural studies. **a,** The similar peak positions shown in SEC analysis confirmed that the truncated SOS1 protein (SOS1$^{\Delta998}$) formed a dimer in solution similar to full-length SOS1 (SOS1^{FL}). **b,** SDS-PAGE analysis with both the full-length and truncated SOS1 protein indicated that the structural integrity of the truncated protein was not compromised. The experiments were repeated independently for more than 3 times with identical results. **c,** Representative motion-corrected cryo-EM micrograph of the truncated protein SOS1$^{\Delta998}$ (Bar = 50 nm) from 1,312 micrographs. **d,** 2D class averaged cryo-EM images comparison in which the intracellular region of SOS1$^{\Delta998}$ is completely blurred and indistinguishable. Source data are provided as a Source Data file.

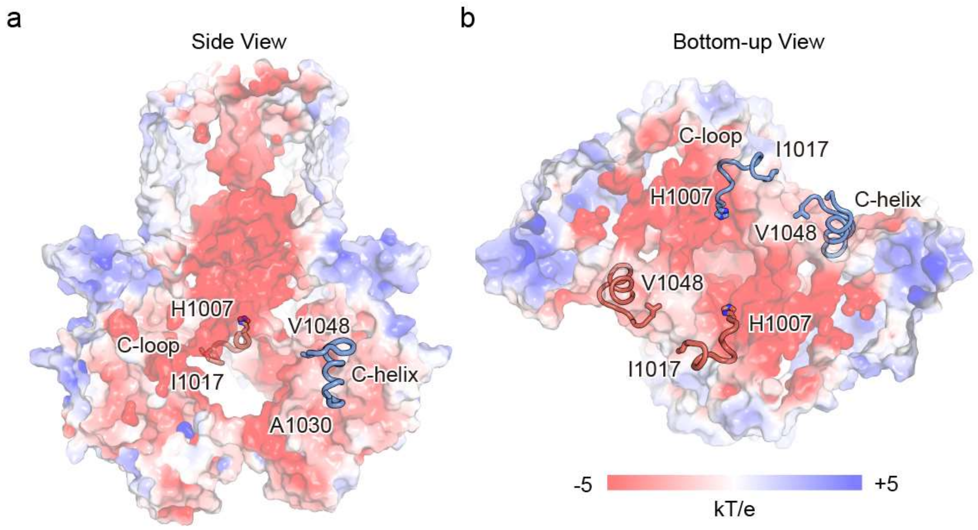

Supplementary Figure 14. Electrostatic potential surface representation of SOS1. Two cross sections from the side view (**a**) and bottom-up view (**b**) through the C-loop and C-helix (cartoon) indicate that the long cytoplasmic tail is located within the negatively charged inner surface, providing the structural basis for the activation mechanism of electrostatic repulsion. Residues at both ends are showed in sticks.

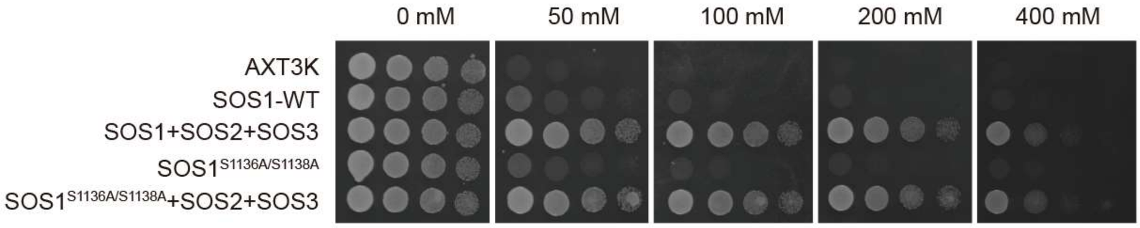

Supplementary Figure 15. Activation of the serine-to-alanine mutant SOS1$^{S1136A/S1138A}$ by SOS2-SOS3 complex. Transgenic yeasts expressing SOS1 and its mutant SOS1$^{S1136A/S1138A}$ were grown in AP medium supplemented with different concentrations of NaCl as indicated, with and without the presence of the SOS2-SOS3 complex.

Supplementary Table 1. Cryo-EM data collection, refinement and validation statistics.

	SOS1 (EMDB-33592) (PDB 7Y3E)	SOS1^{expand} (EMDB-35085) (PDB 8HYA)
**Data collection and processing**		
Magnification	105,000 ×	105,000 ×
Voltage (kV)	300	300
Electron exposure (e–/Å^2)	60	60
Defocus range (μm)	−1.2 ~ −2.2	−1.2 ~ −2.2
Pixel size (Å)	1.04	1.04
Symmetry imposed	C2	C2
Initial particle images (no.)	1,254,798	1,254,798
Final particle images (no.)	146,538	31,721
Map resolution (Å)	2.8	3.4
FSC threshold	0.143	0.143
Map resolution range (Å)	2.7 ~ 3.1	3.3 ~ 3.8
**Refinement**		
Initial model used (PDB code)	7DSX	7Y3E
Model resolution (Å)	3.1	3.8
FSC threshold	0.5	0.5
Map sharpening *B* factor (Å^2)	−94.0	−82.5
Model composition		
Non-hydrogen atoms	14576	14588
Protein residues	1858	1856
Ligands	4	6
B factors (Å^2)		
Protein	42.99	106.36
Ligand	13.83	77.08
R.m.s. deviations		
Bond lengths (Å)	0.004	0.004
Bond angles (°)	0.698	0.715
Validation		
MolProbity score	2.00	2.02
Clashscore	11.98	13.04
Poor rotamers (%)	0.00	0.00
Ramachandran plot		
Favored (%)	93.95	94.12
Allowed (%)	6.05	5.88
Disallowed (%)	0.00	0.00

Supplementary Table 2. Primers used for mutagenesis of SOS1. Bold indicates sequence varying from amplified template.

Primer	Sequence	Remark
D201A	5'-GATGAATG**c**TGGGACGGCGATTGTT-3'	mutation
	5'-CGCCGTCCCA**g**CATTCATCAGGGATT-3'	
W1013A	5'-GTCTCATGAGC**gc**GCCTGAAAATATTTACGCCAAA-3'	mutation
	5'-ATATTTTCAGGC**gc**GCTCATGAGACCTCTGTGTTC-3'	
F1042A	5'-AATGCAACTCAGCATT**gc**CGGCAGCATGGTTAATG TGTAC-3'	mutation
	5'-ACCATGCTGCCG**gc**AATGCTGAGTTGCATTGCTCG-3'	
^{702}EEK^{704}/AAA	5'-GTTGGCTTGTTG**gcggcagcc**GAAATCGCTCATCTTCA TGATGCTG-3'	mutation
	5'-GAGCGATTTC**ggctgccgc**CAACAAGCCAACCTTCTCG AG-3'	
^{484}EEKK^{487}/AAAA	5'-TTCAAGATCTAGGA**gccgctgcggca**CTAGGACCTGCTG ACTGGCC-3'	mutation
	5'-TCCTAG**tgccgcagcggc**TCCTAGATCTTGAAACGCTCG TAAG-3'	
S1136A/S1138A	5'-AT**g**CTCCG**gc**TAAAATCGTTTTCAGGAACGATCTA-3'	mutation
	5'-TGAAAACGATTTTA**gc**CGGAG**c**ATCGATTCTCACAAC GATTCCTT-3'	
ΔC-loop	5'-TCATTTCGTAAAGAATACGCCAAACAACAACAAGA-3'	Internal deletion
	5'-TGTTGTTTGGCGTATTCTTTACGAAATGATCTCTG-3'	
ΔC-helix	5'-AATAAAACGACATACAGAAGGAGTGTAAGTTTC-3'	Internal deletion
	5'-TACACTCCTTCTGTATGTCGTTTTATTGATCTCTT-3'	
Δ998	5'-ACACCACCACGTAGCTCAAGCTCTGATCAGGGTCAT CATCATCATCATCATTGAGG-3'	truncation
	5'-ATCTTGGTACCTCAATGATGATGATGATGATGACCCT GATCAGAGCTTGAGCTAC-3'	

Supplementary Table 3. Determination of Na^+ content by atomic absorption spectrometer.

Na^+ content (mg/L)		Control			30 mM		
	DW	1	2	3	1	2	3
Blank	0	1.208	1.132	1.127	1.208	1.132	1.127
AXT3K	0.01g	2.045	1.975	2.075	17.778	17.818	17.909
SOS1-WT	0.01g	1.925	2.05	1.97	11.855	11.465	11.68
SOS1+SOS2+SOS3	0.01g	1.97	2.01	2.035	9.425	11.195	9.971
ΔC-loop	0.01g	2.235	1.925	2.115	5.91	7.07	6.623
W1013A	0.01g	2.035	1.97	1.93	6.8	5.83	6.815
ΔC-helix	0.01g	2.055	2.04	2.14	10.905	12.205	11.074
F1042A	0.01g	2.05	2.06	2.11	12.225	11.76	10.765
ΔC-loop/C-helix	0.01g	1.935	1.95	1.925	5.215	4.44	4.84
Δ998	0.01g	1.89	1.94	1.755	8.435	8.31	8.401
^{702}EEK^{704}/AAA	0.01g	2.915	2.697	2.689	15.859	15.03	15.577
^{484}DDEE^{487}/AAAA	0.01g	2.173	2.76	2.54	15.721	14.371	14.277

Samples volume was 10 ml.
DW: dry weight.
